# Flagged for Fraud: Lessons From 3 Case Studies on Detecting Inauthentic Participants in Online Research

**DOI:** 10.2196/78554

**Published:** 2025-10-10

**Authors:** Jordan R Hill, Sydney Hoel, Clover Caldwell, Matthew Zuraw, Christian Elliott, Andrew C Pickett, Beth E Fields, Nicole E Werner

**Affiliations:** 1 Department of Health & Wellness Design School of Public Health-Bloomington Indiana University Bloomington, IN United States; 2 Vanderbilt University Medical Center Nashville, TN United States; 3 Whiplash Technology, Inc San Diego, CA United States; 4 Department of Kinesiology University of Wisconsin–Madison Madison, WI United States

**Keywords:** research fraud, online research, participant deception, qualitative study, case study

## Abstract

As digital and remote research methods become more prevalent, the risk of fraudulent participants—individuals who deliberately misrepresent themselves to gain access to studies and associated incentives—has emerged as a significant challenge. These inauthentic participants threaten data validity, obscure treatment effects, and may lead to interventions being developed based on inaccurate representations of target populations. Despite the growing recognition of this issue, researchers have limited guidance on how to detect and respond to fraud when it occurs, particularly when committed by real people rather than automated systems. We present 3 case studies from our own research where participants engaged in deception to gain study incentives. We identify recurring patterns of behavior as “red” (clear signs of inauthenticity) and “yellow” (ambiguous behavior common among fraudulent participants) flags, describe how our team responded, and share lessons learned for future studies. This work aims to support researchers in identifying fraudulent participants more effectively, helping to ensure the validity and credibility of data collected in online research.

## Introduction

To develop effective interventions that enhance human health and wellness, it is crucial to engage human participants in research to identify a target population’s needs and behaviors, as well as to test the efficacy and implementation of developed interventions [1]. The validity of data collected from human participants relies on their membership within the population of interest.

Unfortunately, the issue of fraudulent participants—human participants who falsely claim eligibility for a study or participate multiple times to gain financial incentives [2]—is a documented and growing problem, particularly in studies where all activities are conducted remotely (eg, online surveys and virtual interviews) [2-13]. Pozzar et al [8] have highlighted the potential significance of this issue: their online survey targeting American adults with ovarian cancer received 576 responses within just 7 hours of promotion on social media, with 271 responders self-reporting as meeting the eligibility criteria. Upon investigation, the research team found that 100% of these responses were either fraudulent (94.5%) or suspicious (5.5%).

Including data from fraudulent participants can have significant negative impacts on research, particularly when such responses are unknowingly included in analyses and presented as valid. As described in the study by Chandler et al [9], participant fraud will make it harder to accurately estimate treatment effects in experimental studies, having potential to significantly impact observed relationships within and across groups. By definition, fraudulent responses also do not reflect the values and viewpoints of the population of interest—at best, they reflect external beliefs about this group, which are then erroneously attributed to the target population [9]. These effects become even more pronounced when studies target rare or underrepresented participant groups. Finally, fraudulent responses may also include significant amounts of noise, for example, when surveys are completed by simply filling random responses [14]. In such cases, true experimental effects may be masked, and analyses may be erroneously reported as statistically nonsignificant [13,14].

If an intervention is developed based on the expressed needs or desires of fraudulent participants, it is less likely to be acceptable, useful, or effective for the population for which it was originally intended. Other negative outcomes include the waste of research funding and researcher time [8,15]. Simply being aware of the risk of fraud in a study impacts researcher time because it necessitates the review and verification of all study responses and participants.

As more studies leverage virtual methodologies [16], this problem is only expected to grow, and it is reasonable for researchers to be cautious about fraud when using methodologies that require minimal verification of participants’ identities. It is essential that fraudulent participants are identified and excluded from studies.

There are various types of fraudulent participants in research, and strategies to identify and exclude them differ by type. One type is automated software agents, commonly called “bots,” which imitate human behavior to perform specific tasks independently [17]. Bots are more prevalent in large-scale survey studies that involve many participants and few, if any, direct interactions between researchers and study participants. Bots have been a concern in internet security for many years, and several prevention measures have been developed, including using a completely automated public Turing test to tell computers and humans apart (CAPTCHA) to weed out automated responses, honeypot questions (questions not visible to the human eye but would be seen and answered by bots), and identity verification tools, as well as reviewing survey completion time (as bots will finish a survey much faster than human respondents), identifying identical responses from multiple respondents, performing geolocation checks, avoiding survey promotion on public forums (eg, social media), and embedding specific validation steps [2,6-8,10,11,13].

Another type of fraudulent participant includes individuals who falsely claim eligibility for a study and complete study procedures to gain participation incentives. While this form of fraud is less “efficient” than a bot, it can be more challenging for researchers to identify and prevent these individuals from participating. As these participants are human, tools such as CAPTCHAs and honeypot questions do not filter them out, and direct interactions with study personnel may not reveal their fraudulent status, as they can engage convincingly in real time with the research team. Moreover, widely available AI tools and large language models (eg, ChatGPT) can generate text or responses that mimic those of other individuals [18]; for example, someone wishing to commit fraud by posing as a member of a different demographic group (eg, pretending to be a health care professional to meet eligibility criteria) can use these tools to more convincingly persuade researchers of their legitimacy.

Smaller-scale studies addressing the issue of fraudulent participants have offered recommendations such as interacting with participants nonanonymously (eg, over the telephone), sending financial incentives via traditional mail, and reviewing participant responses and interactions for inconsistencies [2,5,11,12,19].

In conducting 3 studies of our own, we encountered fraudulent participants and found that not only were they difficult to recognize but also that guidance in the literature on identification strategies was limited. Our objective is to address this gap in fraud identification literature by describing “red” and “yellow” flags in participant behavior and interaction that research teams can be aware of when screening participants for inclusion in research studies.

### Case Studies

#### Overview

In this section, we present 3 research studies in which participant fraud was identified. Each case study outlines how the research design was vulnerable to participant fraud, how the fraud was discovered, and how the team responded. Table 1 provides a summary of the designs. All 3 studies were conducted entirely online.

**Table 1:** Summary of the case studies in which participant fraud was identified.

Study	Population of interest	Sample size, n	Study activities	Compensation	Recruitment sources
ADRD^a^ Systematic Hospital Inclusion Family Toolkit (A-SHIFT)	Hospital clinicians and primary caregivers of individuals living with ADRD	27 clinicians and 15 caregivers	Virtual interview and 2 surveys	US $100 total in the form of electronic gift cards	Postings to caregiver and clinician email registries, ADRD community organization promotion (websites and social media posts), and physical flyers posted in community and hospital locations
Resource connection project	Rural primary caregivers of individuals living with ADRD	15	Web-based intervention pilot test, 4 surveys, and virtual interview	US $100 total in the form of electronic gift cards	Postings to caregiver email registries and ADRD community organization promotion (websites and social media posts)
Legal and financial planner	Primary caregivers of individuals living with ADRD	99	Web-based intervention pilot test, 3 surveys, and virtual interview	US $150 total in the form of electronic gift cards	Postings to caregiver email registries and ADRD community organization promotion (websites and social media posts)

^a^ADRD: Alzheimer disease and related dementias.

#### Case Study 1: Alzheimer Disease and Related Dementias Systematic Hospital Inclusion Family Toolkit

##### Overview

The objective of the Alzheimer disease and related dementias (ADRD) Systematic Hospital Inclusion Family Toolkit study was to develop a toolkit to support the inclusion of dementia caregivers in hospital care [20]. In the study, we conducted virtual interviews with dementia caregivers, persons living with dementia, and hospital clinicians. After the interviews, we asked participants to complete online surveys ranking the significance and modifiability of factors influencing dementia caregiver inclusion as identified from the interviews.

##### Recruitment

Recruitment materials contained information about study activities, eligibility criteria, compensation, and research team contact information. Recruitment methods included distributing materials through email lists of professional organizations and societies, hospital systems, and ADRD caregiver research registries; displaying study flyers in physical locations; posting on public social media; and sharing recruitment materials with clinicians and community organizations such as older adult centers, local chapters of the Alzheimer’s Association, and Alzheimer’s Disease Research Centers.

##### Screening and Enrollment

Interested individuals were invited to complete a telephone screening or complete the screening through a Zoom (Zoom Video Communications, Inc) audio call on request.

Caregivers were eligible to participate if they were (1) a current or former primary caregiver to a person living with dementia who had experienced at least 1 hospitalization, (2) aged ≥18 years, and (3) able to speak English. Persons living with dementia were eligible to participate if they were (1) aged ≥65 years and (2) able to speak English. Clinicians were eligible to participate if they (1) had at least 5 years of experience working in a hospital setting and (2) were able to speak English. After screening, eligible individuals reviewed the study information sheet over the telephone (or by Zoom, if requested) with a member of the research team and provided verbal consent.

##### Study Activities

Study activities included completing a virtual 1-hour interview and 2 online surveys.

##### Ethical Considerations

Participants received US $100 in the form of electronic gift cards for completing all study activities (US $50 for the interview and US $25 for each survey). This study was approved by the Institutional Review Board (IRB) at the University of Wisconsin-Madison (approval no. 2022-0024).

##### Cases of Fraudulent Participants

No fraud was suspected or identified among caregiver participants. However, in the clinician group, among those screened, deemed eligible, and enrolled, 3 participants were found to be fraudulent. Fraud 1 stated that they learned about the study on social media. They completed a telephone screening and claimed to be a neurologist with 20 years of experience. Fraud 2 initially contacted the research team via telephone and claimed during the telephone screening to be a junior administrator at a hospital. Fraud 3 requested to complete screening via Zoom and during this session claimed to be a psychiatrist at a teaching hospital with 5 years of experience. All 3 individuals had their cameras switched off during their virtual interviews, making it difficult for the interviewers to verify their identity. Suspicion of fraud arose after these participants completed the first online survey and provided demographic data that conflicted with the information shared in baseline surveys and during interviews. On reviewing the interview transcripts, the lead investigators confirmed that these individuals were not clinicians: their responses were either too vague or included claims about roles and organizations that were not consistent with how care or clinical roles are structured in the health care system. Consequently, all 3 individuals were withdrawn from the study, with the research team citing discrepancies in responses and a subsequent threat to data quality and credibility as the reason for withdrawal.

#### Case Study 2: CareVirtue Resource Connection Pilot Study

##### Overview

The CareVirtue resource connection project aimed to develop a web-based intervention to support rural caregivers of persons with ADRD. After co-designing the intervention with caregivers and representatives of community organizations serving rural ADRD caregivers, the study sought to pilot-test a prototype to obtain user feedback.

##### Recruitment

The two primary recruitment methods were (1) sharing recruitment methods with community organizations that serve ADRD caregivers and (2) distributing recruitment materials via email to members of a caregiver registry. Recruitment materials included contact information for a member of the research team, and interested care partners were instructed to self-identify by contacting the team member. At least 1 public social media post was published on Facebook by a community organization.

##### Screening and Enrollment

Interested individuals were invited to schedule a telephone screening with a member of the research team. To meet inclusion criteria for the study, individuals had to (1) self-identify as a primary caregiver for someone living with ADRD, (2) self-identify as living in a rural area, (3) be aged ≥18 years, and (4) have access to the internet. Individuals who met the eligibility criteria reviewed the study information sheet over the telephone immediately after screening and gave verbal consent to participate.

##### Study Activities

All study activities were conducted virtually. Participants completed an onboarding meeting over Zoom or over the telephone, at which they were given access to the study intervention and completed the registration process and a needs assessment. Next, they were instructed to engage with the intervention for 30 days. During this time, they received recommendations for local and online resources once a week, and after day 7 of the study, they were asked to complete an online feedback survey once per week. At the end of the 30-day use period, participants completed a postuse interview over Zoom or over the telephone about their experiences and opinions of using the intervention.

##### Ethical Considerations

Participants received a US $25 electronic gift card for completing the first visit. They also received a US $75 electronic gift card for completing the 30 days of the study and the postuse interview. This study was approved by the Indiana University IRB (IRB #20189).

##### Cases of Fraudulent Participants

Of the emails we received, 75 were from individuals whom we determined to be fraudulent. Emails were delivered en masse, and the time of delivery coincided with study recruitment materials being shared in a public Facebook post by an organization that served dementia caregivers. In addition, these emails differed from those sent by previous (genuine) participants in that they were short, strangely worded, vague, and had significant grammatical and spelling errors. Of these 75 individuals, 40 (53%) ceased communication with the research team after being informed that they would be required to complete a telephone screening to determine their eligibility for participation. The remaining individuals (35/75, 47%) were flagged as fraudulent based on several factors, including the timing of their initial emails to the research team, the type and format of their email addresses, the content of their emails, and their inability to provide a landline or mobile phone number.

#### Case Study 3: CareVirtue Legal and Financial Planner Pilot Study

##### Overview

The purpose of this pilot study was to confirm the feasibility, usability, and acceptability of a legal planning and financial management training tool for ADRD caregivers.

##### Recruitment

Study information was shared through several channels, including online caregiver registries (eg, Research Inclusion Supports Equity, funded by National Institute on Aging grant R24AG066599) and community organizations (eg, newsletters as well as public postings on websites and Facebook groups).

##### Screening and Enrollment

Participants contacted the research team by email. Researchers then scheduled a screening call via telephone or Zoom and confirmed that participants (1) were a caregiver of a person with ADRD, (2) were aged ≥18 years, (3) had access to the internet, and (4) had access to a desktop or laptop computer. Individuals who met the eligibility criteria reviewed the study information sheet over the telephone immediately after screening and signed an electronic form to document their consent to participate.

##### Study Activities

Participants completed a 30-minute virtual onboarding meeting at the beginning of the study. At this meeting, they completed a baseline survey that included questions about demographic information and financial and legal planning topics. A researcher then guided them through the process of creating a planner account and gave them a tour of the planner website. After a week of using the planner, participants met with a researcher over Zoom for a 15-minute check-in to make sure that they were not experiencing any technical issues. On day 45 of the study, participants received an invitation to complete a midpoint questionnaire about their experiences using the planner. The 3-month study period ended with a semistructured postuse interview over Zoom in which participants were asked about their experiences and opinions of using the planner. Finally, they were asked to complete a postuse survey immediately after the interview.

##### Ethical Considerations

Participants were paid US $40 for completing onboarding. They also received US $20 for completing the midpoint questionnaire. In addition, they were compensated US $50 for completing the postuse interview and survey and received a US $40 bonus if they completed all study activities (maximum possible compensation=US $150). This study was approved by the Indiana University IRB (IRB #16242).

##### Cases of Fraudulent Participants

A total of 318 potential participants initially expressed interest in the study, of whom 216 (67.9%) were not enrolled (n=63, 29.2% due to suspected fraud; n=153, 70.8% for reasons such as ineligibility, lack of interest in the study, or lack of communication with the study team), while 102 (32.1%) were deemed eligible and enrolled. However, of these 102 participants, 3 (0.2%) were later withdrawn after they were suspected of being fraudulent. After cases of fraud in the previous 2 studies were brought to the research team’s attention, additional detection and verification procedures were put in place. Several individuals requested to complete screening via Zoom, which our team did not allow because it had been requested by fraudulent participants in previous studies. Some suspected fraudulent individuals did reply to the study team when told that they must be screened over the telephone, but the majority ceased contact with the study team after they were informed of this requirement. Those who did agree to be screened over the telephone provided numbers that were determined to be voice over IP (VoIP) numbers, which can display a US area code regardless of the user’s actual location. Three individuals were consented before the enhanced screening procedures were implemented and were later identified as fraudulent. They were subsequently withdrawn before study onboarding. One was identified due to their inability to access the intervention (their browser time zone was not set within the United States), while the others were not able to provide a valid telephone number to continue participation.

#### Red and Yellow Flags for Suspected Fraud

Throughout these 3 studies, we observed patterns of behavior that frequently preceded the identification of a fraudulent participant, some of which raised more suspicion than others. Table 2 summarizes our team’s system for identifying these behavioral patterns and categorizes them as either “red flags” (behavioral patterns that should raise the research team’s suspicions and prompt further investigation of the potential participant) or “yellow flags” (patterns common among frauds but also present in some genuine participants). Where relevant, we also provide verbatim examples of interactions with fraudulent participants and compare them with communications sent by nonsuspicious participants.

**Table 2:** Red and yellow “flags” to identify fraudulent participants.

Flags				Examples of fraudulent participants		Examples of real participants
**Red**						
	Interest email	Short Not signed with person’s name No salutation Significant grammatical errors No subject line, or subject line is a strange study name Email text is identical to email text sent by another interested person (likely also fraudulent) Generic and does not provide detail	No subject; Body: “I am interested to partake in the he survey” Subject: “Interested I take caregiver for mother with dementia;” No body.		Subject: “Dementia Caregiver Study;” Body: “Hello, I received the email from the FTDa Disorders Registry about your study. My husband has bvFTDb and was diagnosed in 2019. I’d like to know if I may participate, and of course, first know more about it. I did read the summary of the criteria and think I meet it. Thank you, [NAME]”	
	Compensation	Overly concerned with study compensation Sends multiple emails in a short period about getting compensated May become aggressive about compensation and demand it (eg, using guilt-inducing language and capitalizing the subject line)	Body: “After completing this study you promised to provide and we will receive a compensation but till date I haven’t seen anything, receiving and email that I have been removed from the study but haven’t been compensate So all this thing aren’t Good I haven’t received my promised compensation and you removed me from the study because of am the last or what please explain And I completed this study long ago that is last year December which is unfair Take it to be like you how will you feel about this? Thank you for your response”		Body (sent 8 days after completing a survey): “Hi [researcher], I completed this survey on 3/11. I wanted to be sure this was reflected in your records. Thank you! [name]”	
	Telephone number	Person refuses to provide a telephone number Person insists on communicating through videoconferencing rather than over the telephone Provided telephone numbers are VoIPc numbers (vs landline or mobile phone numbers)	—^d^		—	
	Address and zip code confusion	Zip code provided does not match the address given Takes the person a long time to provide their zip code when asked for it (as if they have to look it up) Address does not exist or is for a nonresidential building (eg, a church)	—		—	
	Diagnosis and condition details	Provides incorrect details or cannot provide specific details on the condition of interest to the study when asked, or it takes a long time for them to answer (eg, care recipient dementia type)	Interviewer: “In the inpatient hospital setting, what would your role be in the admission process?” Respondent: “Yeah. In the admission process, I’m a neurologist, yeah, and also I’m an administrator as well, yeah, regarding my field of specialty because we’re not really much here, so I have a lot of function in there, so.”		Respondent: “I am a caregiver to my spouse, who was diagnosed with frontotemporal dementia with PPAe (logopenic variant) two years ago. He has been living with symptoms for at least six years. I am very interested in participating in this particular program/study.”	
	ID check	When asked to provide ID on camera, person refuses or cannot comply	—		—	
**Yellow**						
	Email address	Uses a Gmail account with the following structure: Firstnamelastname#@gmail.com Email address differs from name provided in the study	Johnsmith1@gmail.com Doejane46@gmail.com These email addresses are illustrative examples created by the authors and were not used by any actual participants (genuine or fraudulent)		—	
	“How did you hear about the study?”	Answer is vague (eg, Facebook, newsletter, or a friend) Friend who referred them to the study is not enrolled in the study or is also a suspected fraud	“I heard about it on social media” “A colleague referred me to study”		“Found your study on the AFTDf website. I am interested in your study. My husband was diagnosed with BVFTD in April 2021. I am his full time caregiver, he lives at home.”	
	Delay in answering questions	For example, date of birth, telephone number, and address Uses a large number of filler words before giving an answer	—		—	
	Videoconferencing behavior	Does not turn on their camera Provides short answers to questions and does not elaborate	—		—	
	Address	Major metropolitan locations (eg, Los Angeles, Miami, or New York City) Generic addresses (eg, [##] W [#]th St; [###] Main St) Incomplete addresses provided When reading their address aloud, they pronounce it strangely	—		—	

^a^FTD: frontotemporal dementia.

^b^bvFTD: behavioral variant frontotemporal dementia.

^c^VoIP: voice over IP.

^d^Not applicable.

^e^PPA: primary progressive aphasia.

^f^AFTD: Association for Frontotemporal Degeneration.

We determined that it was necessary to separate behaviors into 2 categories: behaviors that strongly indicated fraud (eg, inability to provide a valid telephone number or providing an incorrect zip code); and behaviors that, while nearly ubiquitous among fraudulent participants, were also exhibited by genuine participants (eg, having a Gmail account or keeping the camera switched off during videoconferencing). We thought that it was important to include the “yellow flags” for additional context for researchers. While the presence of a single flag, particularly a yellow one, does not necessarily mean that a participant is inauthentic (for instance, many legitimate participants use Gmail accounts, live in major metropolitan areas, or exhibit disengaged behaviors), recognizing these behavior patterns can alert the research team to potential fraud and prompt further investigation.

### Strategies to Prevent Fraudulent Participation

Participant fraud is a risk that must be considered and accounted for in the design and conduct of research studies. The simplest way to reduce this risk may be to conduct study activities in person, thereby requiring face-to-face interaction with members of the research team. However, virtual research activities offer significant benefits to both researchers and participants. Remote research facilitates the recruitment of participants who might otherwise be unable to participate by overcoming geographic constraints, minimizing the burden of participation (eg, no need to travel), and enabling individuals who are homebound to take part [21-23]. In the earlier phase of the study, presented as the second case study in this paper, remote study operations enabled the recruitment of rural ADRD caregivers from throughout the United States (none of whom were in the researchers’ home state), which would not have been possible with traditional in-person methods [24]. Decentralization can also lead to cost savings for the researcher [25].

To continue leveraging the opportunities created by virtual research, we as researchers must recognize the elements of study design that increase vulnerability to fraud; implement prevention strategies; and continuously monitor for fraud during recruitment, enrollment, and data collection. The strategies presented in Table 3 stem from the authors’ experiences with participant fraud across the 3 previously described studies and have been reinforced in other fraud prevention publications.

**Table 3:** Suggested strategies according to the study phases to prevent and identify fraudulent participants.

Study phase	Strategies
Recruitment	Use targeted recruitment approaches such as emailing private registries, engaging a liaison with access to the population of interest, posting in newsletters circulated to a limited audience, or contacting potential participants directly When possible, post physical flyers where potential participants congregate Avoid public websites or social media (eg, Facebook) posts Avoid specifying compensation amounts in public postings Ask participants where they heard about the study; press for details if they provide vague responses
Screening and enrollment	Verify information provided by participants Telephone number: use a phone number validation tool to determine if the participant is using a voice over IP number Mailing address: check online to determine whether an address is valid Personal data: have participants reconfirm personal data (eg, date of birth) and look for discrepancies Perform screening and enrollment over the phone instead of using videoconferencing software Require an ID check from the participant through the webcam (no identifiable information from the ID check needs to be recorded; simply verify the individual’s name) if videoconferencing is necessary Order questions to “catch” frauds (eg, ask where they live, then ask for the zip code, and note whether they have difficulty answering or whether the zip code matches the address) Ask for details about the condition of interest when applicable and note whether they have difficulties answering, for example, the type of dementia or specific duties of a clinician in a clinical role
Data collection	Implement location restrictions, if possible, when testing a digital intervention (eg, through IP address and browser time zone) Note whether the participant’s camera is switched off during study interactions over videoconferencing Note erroneous statements that a legitimate participant would not make (eg, a clinician expecting their patient with dementia to “get better”) Ask participants to resubmit demographic data in multiple surveys and check for discrepancies

## Discussion

### Summary

On the basis of 3 case studies, this paper presents a “red” and “yellow” flag framework for identifying individuals falsely claiming to be eligible for a study. We noticed suspicious behaviors among suspected frauds that were also published in other papers, such as keeping cameras switched off during videoconferencing [2,12,19], not providing a valid US telephone number [2,5,11], having a Gmail account or email address with a specific structure [5,12], short interest emails lacking contextual information [19], providing nonresidential addresses as home addresses [11], inability to provide specific details on the condition of interest or providing details that do not align with the stated condition [5,19], vague responses when asked how they were referred to the study [5], and unusual pauses or delays when answering questions [19]. However, we believe that some of the behaviors identified in these 3 case studies have not yet been documented in the literature, such as the prevalence of VoIP numbers, strange pronunciations of addresses, and the prevalence of addresses in large metropolitan areas (eg, New York City). Our study also categorized these behaviors by severity, indicating whether something was a strong indication that a participant was fraudulent (“red flag”) or whether it was also a behavior exhibited by genuine participants but was particularly common among frauds (“yellow flag”), which is novel and expands on previous work.

Similarly, we provide a series of recommendations to prevent fraudulent participation in online qualitative research studies such as our 3 case studies. Some of these—such as implementing location restrictions using IP addresses [2,5,12,19], limiting compensation details in public postings [5,19], avoiding public study advertising (eg, on social media) [19], and verifying participant identity (eg, demographic, ID, or address checks) [19]—have already been documented in the literature while others (eg, ordering questions to “catch” frauds) have not been as prominently discussed.

Fraudulent research participants create multiple issues beyond compromising data quality and rigor. Research staff in studies with smaller samples now spend extra time and resources investigating every potential participant, a task that may be infeasible in studies with large samples. If a large number of frauds are enrolled, recruitment may need to be paused or delayed to allow research staff to identify and exclude them. Incentives for participation may be awarded to frauds instead of genuine participants, detracting from fixed project budgets allocated for participant compensation and wasting resources often funded by the government and taxpayers.

Perhaps the greatest disservice caused by frauds is to the genuine individuals who volunteer their time, accept the risks of participating in a research study, and provide data that enable scientific advancement. Additional steps to verify an individual’s identity increase the burden on genuine research participants. Many signs of participant fraud are “yellow flags” with multiple interpretations, creating the risk that genuine individuals will be turned away or withdrawn from research for appearing too similar to frauds; for instance, a VoIP number may be used by a fraudulent individual or by a caregiver because it is less expensive than a mobile phone contract. Likewise, free email accounts such as Gmail are widely used and convenient, making them accessible to both fraudsters and genuine participants (including those who may not have paid or institution-based email accounts). We want to clarify that we do not recommend excluding someone from study participation based on a single “flag” but rather advocate for considering all contextual information to make the best decision. The need for research staff to make a judgment call on including a participant raises ethical concerns regarding biases. For this reason, we suggest incorporating fraud prevention measures (eg, requiring study screening to be conducted over the telephone, not over VoIP numbers) into the study’s standard operating procedures to ensure that all study participants are held to the same standard. While this may inadvertently exclude some authentic study participants, this is a risk the study team must evaluate against the need to recruit a legitimate study sample.

Conversely, there are also ethical issues associated with failing to take appropriate steps to remove fraudulent participants from research samples. Individuals who assert membership in the study population may misrepresent the experiences, perspectives, and opinions of actual members of this group; for instance, a fraudulent participant in the ADRD Systematic Hospital Inclusion Family Toolkit study (case study 1) claimed that their hospital had an abundance of resources for family caregivers. If undetected, they might have created the false impression that clinicians possess the resources needed to adequately involve caregivers in hospital care. In our experience, many of these fraudulent participants identify as members of sociodemographic groups that are underrepresented in research (eg, Black or African American), which could lead to misleading assumptions about the needs, beliefs, or characteristics of these groups and further undermine their trust in research.

Balancing the competing risks of being too “strict” and excluding genuine participants with being too “lax” and failing to catch fraudulent ones is a challenge without a clear solution that perfectly fits every research team, study, or participant population. Research teams must work to understand the risks associated with fraud, consider strategies for mitigation and their associated costs, and then make an appropriate determination for their specific circumstances. This may mean adjusting analyses, allocating more funding in budgets to recruiting and screening efforts, and acknowledging the risk of fraudulent participation in limitations sections in publications.

It is important to note that the strategies recommended in this paper and the conclusions drawn are based solely on the authors’ experiences with the 3 aforementioned studies. The studies had differing designs and used various recruitment strategies but all had relatively small sample sizes and involved in-depth interactions with the study team (eg, virtual interviews). We believe that our flag framework and mitigation strategies will be most useful to researchers conducting studies with similar characteristics: (1) sample sizes small enough to allow enhanced screening by the research team, (2) real-time interactions between researchers and participants to judge authenticity, or (3) data collection at multiple time points in the study to compare answers to check for discrepancies.

Studies that rely on large sample sizes (eg, hundreds to thousands of participants) and collect data exclusively through online surveys may encounter unique challenges related to participant fraud. Some strategies suggested in this paper may not be applicable to large samples; for example, online surveys may be more susceptible to bots than to fraudulent participants, necessitating different strategies to address these issues (eg, CAPTCHAs and honeypot questions).

Finally, there is no definitive way to ascertain whether we detected all instances of fraud in the 3 aforementioned studies or whether we mistakenly identified any genuine individuals as fraudulent and erroneously declared them ineligible. Our inability to objectively determine whether a participant truly was fraudulent means that we cannot assess the effectiveness of our strategies.

### Conclusions

Fraudulent participants pose an increasingly prevalent challenge in virtual research. It is essential to remove such individuals from study samples to uphold the integrity of the research and avoid harming relationships with genuine participants. In this paper, we have presented behavioral patterns and strategies to identify and prevent fraudulent research participation based on our experiences with 3 different studies. We believe that these recommendations are valuable to others conducting similar research, while acknowledging that there is no one-size-fits-all solution to addressing this issue. Research teams must balance the competing interests of eliminating frauds and ensuring that a diverse array of individuals can participate in research with minimal burden.

## Abbreviations

ADRD: Alzheimer disease and related dementias
CAPTCHA: completely automated public Turing test to tell computers and humans apart
VoIP: voice over IP

## Data Availability

Data sharing is not applicable to this paper as no datasets were generated or analyzed during this study.
